# Does body mass index affect restoration of femoral offset, leg length and cup positioning after total hip arthroplasty? A prospective cohort study

**DOI:** 10.1186/s12891-019-2790-y

**Published:** 2019-09-12

**Authors:** Bariq Al-Amiry, Georgios Pantelakis, Sarwar Mahmood, Bakir Kadum, Torkel B. Brismar, Arkan S. Sayed-Noor

**Affiliations:** 1 Department of Surgical and Perioperative Sciences, Umeå University, 901 85 Umeå, Sweden; 2 Institutionen för klinisk och experimentell medicin, Linköping University, 58183 Linköping, Sweden; 3 Department of Clinical Science, Intervention and Technology, Karolinska Institute, 171 77 Stockholm, Sweden

**Keywords:** BMI, Hip arthroplasty, Femoral offset, Leg length discrepancy, Cup positioning

## Abstract

**Background:**

In obese patients, total hip arthroplasty (THA) can be technically demanding with increased perioperative risks. The aim of this prospective cohort study is to evaluate the effect of body mass index (BMI) on radiological restoration of femoral offset (FO) and leg length as well as acetabular cup positioning.

**Methods:**

In this prospective study, patients with unilateral primary osteoarthritis (OA) treated with THA between September 2010 and December 2013 were considered for inclusion. The perioperative plain radiographs were standardised and used to measure the preoperative degree of hip osteoarthritis, postoperative FO, leg length discrepancy (LLD), acetabular component inclination and anteversion.

**Results:**

We included 213 patients (74.5% of those considered for inclusion) with a mean BMI of 27.7 (SD 4.5) in the final analysis. The postoperative FO was improper in 55% and the LLD in 15%, while the cup inclination and anteversion were improper in 13 and 23% of patients respectively. A multivariable logistic regression model identified BMI as the only factor that affected LLD. Increased BMI increased the risk of LLD (OR 1.14, 95% CI 1.04 to 1.25). No other factors included in the model affected any of the primary or secondary outcomes.

**Conclusion:**

Increased BMI showed a negative effect on restoration of post-THA leg length but not on restoration of FO or positioning of the acetabular cup. Age, gender, OA duration or radiological severity and surgeon’s experience showed no relation to post-THA restoration of FO, leg length or cup positioning.

## Background

Total hip arthroplasty (THA) is a cost-effective and successful surgical intervention for patients with hip osteoarthritis (OA) complaining of persistent pain and disability [1]. Apart from alleviating pain and improving function and quality of life, THA aims to restore the biomechanical forces around the hip with appropriate femoral offset (FO) and leg length [2–4]. Failure to restore FO, for instance, might result in worse functional outcome, and prosthetic instability while post-THA leg length discrepancy (LLD) can give rise to patient dissatisfaction, limping, gait disorders and increased use of shoe lifts [5–10]. Furthermore, inadequate positioning of the acetabular cup may be associated with impingement and prosthetic dislocation [11].

The prevalence of obesity among children and adults is increasing worldwide [12]. In obese patients undergoing THA, the thick fatty tissue may obscure bony landmarks, deteriorate optimal implant positioning and prolong operative time. The effect of body mass index (BMI) on THA functional outcome, quality of life and complication rate has been investigated in a number of clinical studies [13–16]. As BMI increases, the functional improvement and quality of life after THA may deteriorate and the rate of postoperative complications increases [17]. A number of studies have also investigated the relation between BMI and cup positioning and showed contradictory results [18–20]. Nevertheless, there is paucity of knowledge in regard to how BMI can affect the restoration of FO and leg length after THA.

The aim of this prospective cohort study is to evaluate the effect of BMI on post-THA radiological restoration of FO and leg length as well as acetabular cup positioning. We hypothesized that BMI would increase the risk for improper radiological restoration of FO and leg length as well as acetabular cup positioning.

## Methods

Between September 2010 and December 2013, patients with radiological symptomatic unilateral primary OA treated with THA due to conservative treatment failure were considered for inclusion. Exclusion criteria were secondary OA, previous vertebral, pelvic, or lower limb fractures or surgeries. At the outpatient’s visit before the operation, we documented each patient’s BMI (weight (kg) / [height (m)]^2^) and the duration of OA symptoms as less or longer than 3 years. As per our department’s routine, preoperative plain radiographs were adequate for the operation if they were taken within 3 months preoperatively, to measure the degree of radiological OA [Kellgren-Lawrence (KL) classification, divided into 2 categories: mild OA (KL 1–2) and severe OA (KL 3–4)] [21].

The operative approach was the postero-lateral with the patient in the lateral decubitus position. Two THA types were used, cemented Lubinus SP II system (Link, Germany) or cementless Spotorno (CLS) stem and Trilogy cup (Zimmer, USA). The Lubinus stem has a center collum diaphyseal (CCD) angle of 126°, 32 mm head, and 3 neck lengths (47.5, 51.5, and 55 mm). The CLS stem has a CCD angle of 125°, 32 mm head, and 4 neck lengths (− 4, 0, 4, and 8 mm). We used the Mdesk system (RSA Biomedical, Umeå, Sweden) for preoperative templating. However, the final choice of prosthetic component combinations depended on the surgeon’s intraoperative evaluation. Two to three days after the operation, postoperative plain radiographs were taken in supine position and 15° internal rotation of both legs while the X-ray beam centered on the pubic symphysis with a film to focus distance of 115 cm. A calibration 30-mm radiopaque standardized metal sphere (30 mm) was put between the upper thighs to assess the degree of magnification. Acceptable radiographs were visually evaluated in each patient and considered adequate if centred with equally sized obturator foramina. When apparent or suspected difference existed, we used the program to calculate the difference and when more than 10%, new radiographs were ordered (*n* = 8). Radiographs were monitered using the Picture Archiving and Communication System (PACS) (Impax: Agfa, Antwerp, Belgium) on a 19-in. LCD monitor.

**Fig. 1:**
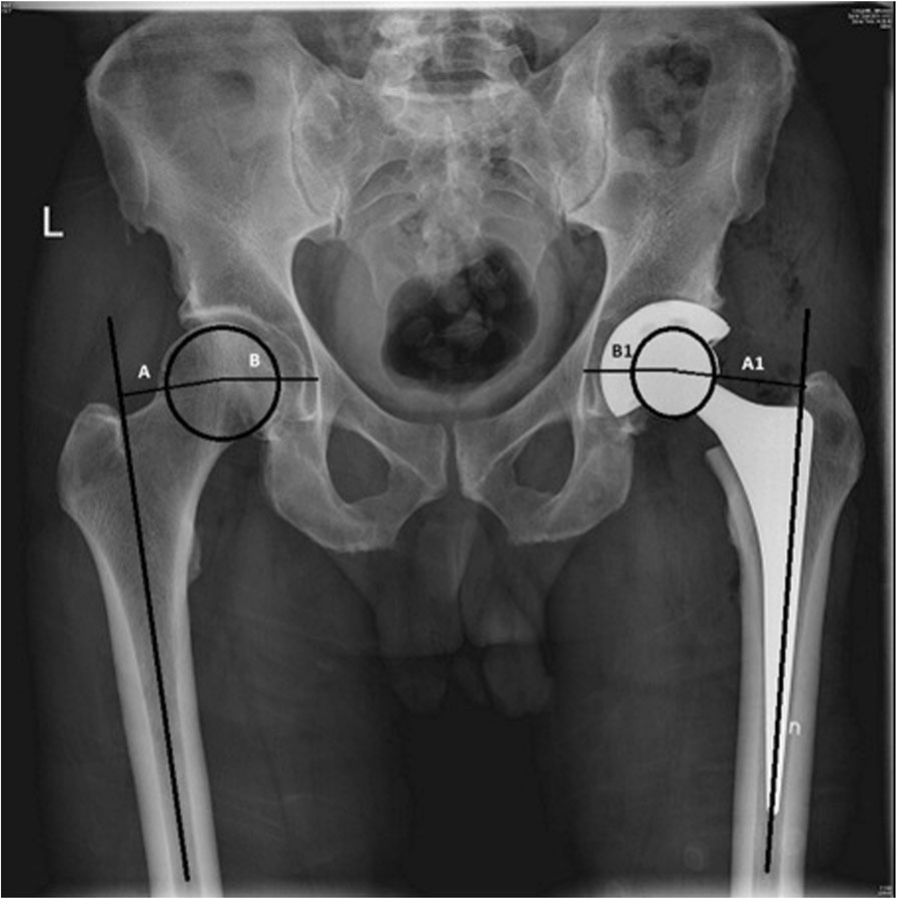
Plain radiograph measurement of the global FO by adding the distance between the longitudinal axis of the femur and the centre of the femoral head (femoral offset) to the distance from the centre of the femoral head to a perpendicular line passing through the medial edge of the ipsilateral teardrop point of the pelvis (cup offset)

The global FO of the THA side was measured as the distance between the longitudinal axis of the femur, at the upper 1/3 to ½ of the diaphysis, where the thickness of the cortices is even, to the center of rotation (stem offset) plus the distance from the center of hip rotation to a vertical line of the medial edge of the ipsilateral teardrop point of the pelvis (cup offset) (Fig. 1), [5]. When within 5 mm compared to the contralateral healthy side, the THA hip FO was considered proper. If it was less or more than 5 mm, the THA hip FO was considered improper.

**Fig. 2:**
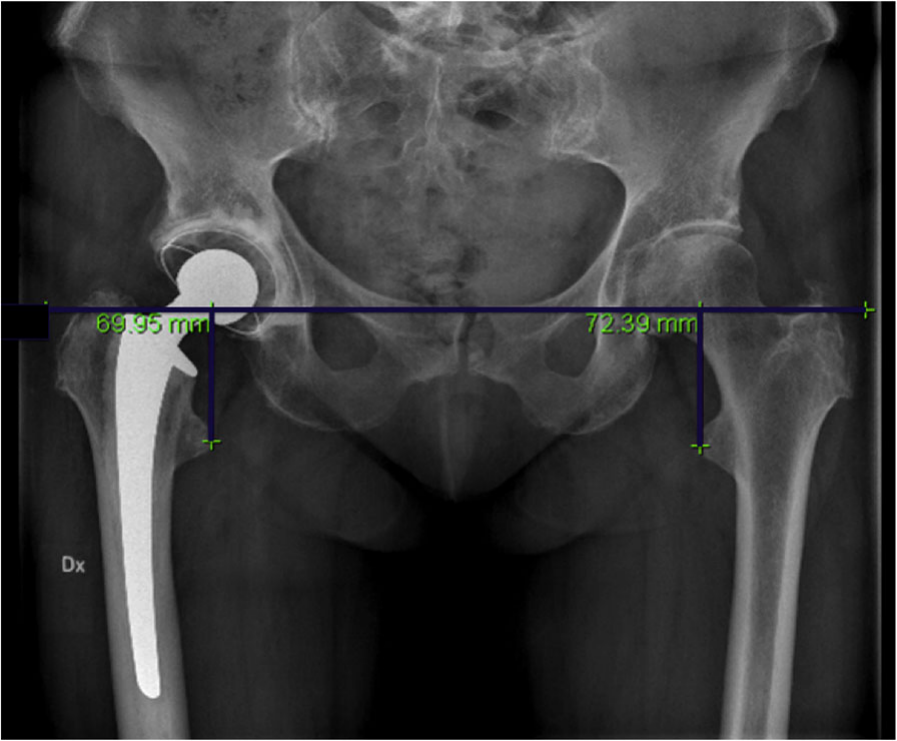
Plain radiograph measurement of the leg length discrepancy as the perpendicular distance between a line passing through the lower edge of the teardrop points to the corresponding tip of the lesser trochanter

The radiological LLD was calculated as the difference in vertical distance between the lower margins of the teardrop points to the corresponding tips of the lesser trochanters (Fig. 2), [7]. Lengthening or shortening of the THA hip compared to the contralateral healthy side within 10 mm was considered proper. Measures outside this range were considered improper.

Cup inclination was measured on the AP view as the angle between a line of the angle of the cup rim and the line between the lowest points of the ischial tuberosities [22]. Operated cup inclination of 45 ± 10° was considered proper. Measures outside this range were considered improper. Also, cup anteversion was calculated on the lateral radiograph as the angle between a line across the face of the acetabulum and a line perpendicular to the horizontal plane [23]. The THA cup anteversion of 15 ± 10° was considered proper. More or less than this was considered improper.

The clinical research work was conducted in accordance with the Declaration of Helsinki and the regional ethics committee approved it. All patients gave informed consent before participation.

### Statistical analysis

We used the method of Peduzzi et al. to estimate the required sample size and study power [24]. Based on the FO and LLD, considered as our primary outcome measures, with an expected incidence of abnormal outcome of 30% of patients (0.30), the minimum number of patients required was 200, calculated as 10 x number of cofounders (*n* = 6) divided by the proportion of expected abnormal cases (0.30).

A multivariate logistic regression analysis was fitted for each outcome measure to test if there is any cause-effect relationship between BMI and outcome measures. We adjusted this for *priori* confounding factors, including age, sex, surgeon’s experience, KL class, and symptom duration. We chose these factors as we anticipated these to be related both to exposure and outcome, and that they would not be in the causal pathway. The odds ratio (OR) and 95% confidence interval (95% CI) are presented. A *p-*value < 0.05 was considered statistically significant.

## Results

We considered 286 patients for inclusion during the study period. We excluded 21 (7.3%) who had one or more exclusion criteria. Fifteen patients (5.2%) did not agree to participate in the study and 37 patients (13%) had no prospectively measured or documented BMI. This left the analysis with 213 (74.5%) patients. There were 118 females (55%) and 105 males (45%) with a mean age of 68 years (SD 10). The mean BMI was 27.7 (SD 4.5), 60 patients with BMI < 25, 94 patients with BMI between 25 and 29.99 and 59 patients with BMI ≥ 30. Regarding the radiological OA severity, there were 73 patients in the mild OA group and 143 patients in the severe OA group. Regarding symptom duration, there were 97 patients in the group with symptom duration < 3 years and 116 patients in the group with symptom duration > 3 years.

The mean FO in the cohort was − 2 mm (SD 9). There were 118 patients (55%) with improper FO: 73 patients with decreased FO and 45 patients with increased FO.

The mean LLD in the cohort was 2 mm (SD 7). There were 32 patients (15%) with improper LLD: 9 patients with shortening and 23 patients with lengthening.

The mean cup inclination in the cohort was 47° (SD 7). There were 27 patients (13%) with improper cup inclination: 9 patients with increased inclination and 16 patients with decreased inclination.

The mean cup anteversion in the cohort was 17° (SD 8). There were 50 patients (23%) with improper cup anteversion: 32 patients with increased anteversion and 18 patients with decreased anteversion.

The multivariable logistic regression model identified BMI as the only factor that affected LLD (Table 1). Increased BMI increased the risk of LLD (OR 1.14, 95% CI 1.04 to 1.25). No other factors included in the model affected any of the primary or secondary outcomes (Table 2).

**Table 1:** Comparison of the effect of gender, age, BMI, the surgeon’s experience, OA grade and OA symptom duration on the primary outcome measurements, FO and LLD

Femoral-offset restoration	OR	95% CI	*p*-value
Gender			
Male	1.00	Ref	
Female	0.70	0.39 to 1.27	0.24
Age	0.99	0.96 to 1.02	0.71
BMI	1.01	0.95 to 1.01	0.71
Surgeon			
Consultant	1.00	Ref	
Resident	1.56	0.79 to 3.10	0.19
OA grade			
K-L grade 1–2	1.00	Ref	
K-L grade 3–4	0.90	0.50 to 1.60	0.72
OA duration			
< 3 years	1.00	Ref	
≥ 3 years	0.73	0.40 to 1.33	0.31
Leg length discrepancy	OR	95% CI	*p*-value
Gender			
Male	1.00	Ref	
Female	0.66	0.28 to 1.54	0.34
Age	0.99	0.95 to 1.05	0.88
BMI	1.14	1.04 to 1.25	**0.005**
Surgeon			
Consultant	1.00	Ref	
Resident	0.90	0.33 to 2.44	0.84
OA grade			
K-L grade 1–2	1.00	Ref	
K-L grade 3–4	0.75	0.33 to 1.69	0.49
OA duration			
< 3 years	1.00	Ref	
≥ 3 years	0.94	0.41 to 2.20	0.89

**Table 2:** Comparison of the effect of gender, age, BMI, the surgeon’s experience, OA grade and OA symptom duration on the secondary outcome measurements, cup inclination and anteversion

Cup inclination	OR	95% CI	*p*-value
Gender			
Male	1.00	Ref	
Female	1.18	0.50 to 2.75	0.71
Age	0.98	0.94 to 1.03	0.41
BMI	1.02	0.93 to 1.16	0.67
Surgeon			
Consultant	1.00	Ref	
Resident	1.11	0.42 to 2.94	0.84
OA grade			
K-L grade 1–2	1.00	Ref	
K-L grade 3–4	1.17	0.51 to 2.68	0.70
OA duration			
< 3 years	1.00	Ref	
≥ 3 years	1.68	0.68 to 4.14	0.26
Cup anteversion	OR	95% CI	*p*-value
Gender			
Male	1.00	Ref	
Female	1.10	0.55 to 2.18	0.79
Age	0.97	0.94 to 1.01	0.13
BMI	0.97	0.90 to 1.05	0.49
Surgeon			
Consultant	1.00	Ref	
Resident	0.63	0.27 to 1.45	0.28
OA grade			
K-L grade 1–2	1.00	Ref	
K-L grade 3–4	0.88	0.45 to 1.72	0.71
OA duration			
< 3 years	1.00	Ref	
≥ 3 years	0.94	0.47 to 1.88	0.85

## Discussion

This study revealed no effect of BMI on postoperative restoration of global FO or positioning of the cup. However, increased BMI was associated with LLD, mainly lengthening of the operated leg. Age, sex, surgeon’s experience, KL class, and symptom duration did not affect any of the outcome parameters. This study could be the first one in the literature to report the relation between BMI and the restoration of FO and LLD after THA. We chose the above-mentioned confounders because we evaluated old age, high grade OA, long lasting OA and less experienced surgeons to possibly affect the risk of improper FO and leg length restoration and cup positioning. Gender was also considered since anatomical differences between males and females may have influence on the outcomes.

The measurement of FO is an essential perioperative radiological step in THA. Femoral offset is commonly defined as the distance between the femoral head center of rotation and the long axis of the femoral shaft [25]. However, this measurement does not consider the possible changes caused by variations of the cup positioning. This variation can be calculated as the cup offset and defined as the distance between the center of the femoral head and a perpendicular line passing through the medial edge of the ipsilateral acetabular teardrop [26]. The global FO is obtained as the summation of FO and cup offset. In this study, the contralateral healthy hip was used as a reference. We used the 5-mm cut-off to determine the proper from improper FO, because previous reports showed that this value could influence the functional outcome [4, 27, 28]. We anticipated that increased BMI would jeopardies proper FO restoration because of the intra-operative mechanical difficulty caused by the extensive adipose tissue and obscured osseous landmarks. About 55% of our cases had an improperly restored FO. However, none of the included confounders had any effect. In our clinical practice, we do not use any intra-operative method to check for the FO. We think the available methods need to be assessed to prove their validity and reliability. It would be interesting to include such intra-operative methods in future studies to determine their effect on FO restoration [29].

The degree of tolerated LLD after THA varies widely in the literature [30, 31]. Commonly, inadequate femoral neck osteotomy and positioning of the stem result in post THA LLD [32]. Less than 10 mm of postoperative LLD is often considered acceptable by most clinicians. Therefore, we used this cut-off to determine proper from improper restoration. Approximately 15% of our cases had improper LLD, 72% of them with lengthening > 10 mm. To ensure minimal intraoperative LLD, we compare the knee and heel level of the operated leg to the other leg and by applying axial traction on the operated hip to evaluate the tension of the surrounding soft tissues and the jumping distance of the prosthetic head after the insertion of prosthetic trial components. Our results showed that increased BMI was associated with LLD (OR 1.14, 95% CI 0.04 to 0.25, *p* < 0.005). This association could be explained by the intra-operative difficulty in comparing the two legs and assessing the soft tissue tension in obese patients.

The effect of BMI on the acetabular cup positioning has been examined in a number of previous studies. We used the Woo and Morrey method [23] for measuring cup anteversion because it is commonly used in the literature and we had studied its reliability in our material in a previous study [33]. This would also allow us to compare our results with others. We chose the cut-off values for cup inclination of 45 ± 10° and cup anteversion of 15 ± 10° because these values are generally accepted as the proper safe zone positioning for prosthetic stability. However, we are aware of the debate in the literature about the validity of these values [34]. Agreed with our results, Bosker et al._,_ Pirard et al. and Todkar reported no association between BMI and cup anteversion or inclination [17, 35, 36]. Bosker et al. [34], found that patient’s age and surgeon’s experience significantly influenced cup positioning, while Callanan et al_._ revealed that the surgical approach, surgeon volume, body mass index > 30 to independently predict malpositioned cups, both inclination and anteversion [37]. Also, Elson et al. reported a significant correlation between morbid obesity (BMI > 35) and under-anteversion [20]. Of all variables considered, high BMI was the most significant risk factor leading to malpositioning in their study. In a case-control study, Brodt et al. showed that BMI correlated with reduced cup anteversion but not with inclination [38].

The present study has limitations. Plain radiographs can be compromised by alterations in pelvis positioning and the X-ray beams divergence. We used a standardized positioning protocol to ensure correct positioning, even though we could not guarantee this 100%. Also, plain radiographs might underestimate the change in FO and LLD. As we calculated the bilateral differences, we considered this underestimation to be negligible. Computerized tomography (CT) scans would certainly have improved the accuracy of our radiological measurements. However, CT-scans are not suitable as a routine perioperative evaluation method for THA patients, owing to their high cost, limited availability and high radiation dose. Furthermore, the validity and reliability of plain radiographic methods have also been investigated and found to be clinically acceptable [33]. The sample size of this study could be underpowered to elicit an effect of BMI on the relatively low incidence of improper cup positioning. Also, the relatively limited number of obese patients with BMI ≥ 30 (*n* = 59) did not allow us to make further analysis in regard to the influence of different grades of obesity on the studied parameters.

## Conclusion

This study showed that increased BMI had a negative effect on restoration of post-THA leg length but not on restoration of FO or positioning of the acetabular cup. Age, gender, OA duration or radiological severity and surgeon’s experience showed no relation to post-THA restoration of FO, leg length or cup positioning. These results can help THA surgeons to improve their preoperative planning and patient’s information to get the best possible restoration of the operated hip geometry, especially in patients with high BMI where intraoperative measures to correct LLD could be considered.

## Data Availability

All data is stored in the trial registry. And the datasets used or analysed during the current study are available from the corresponding author on reasonable request.

## Abbreviations

BMI: Body mass index
CCD: Center collum diaphyseal
CI: Confidence interval
FO: Femoral offset
LLD: Leg length discrepancy
OA: Osteoarthritis
OR: Odds ratio
PACS: Picture archiving and communication system
THA: Total hip arthroplasty
